# Systematic identification of transcription factors associated with patient survival in cancers

**DOI:** 10.1186/1471-2164-10-225

**Published:** 2009-05-15

**Authors:** Chao Cheng, Lei M Li, Pedro Alves, Mark Gerstein

**Affiliations:** 1 Program in Computational Biology and Bioinformatics, Yale University, New Haven, CT, USA; 2 Molecular and Computational Biology Program, Department of Biological Sciences, University of Southern California, Los Angeles, CA 90089, USA; 3 Department of Mathematics, University of Southern California, Los Angeles, CA 90089, USA; 4 Department of Molecular Biophysics and Biochemistry, Yale University, New Haven, CT, USA; 5 Department of Computer Science, Yale University, New Haven, CT, USA

## Abstract

**Background:**

Aberrant activation or expression of transcription factors has been implicated in the tumorigenesis of various types of cancer. In spite of the prevalent application of microarray experiments for profiling gene expression in cancer samples, they provide limited information regarding the activities of transcription factors. However, the association between transcription factors and cancers is largely dependent on the transcription regulatory activities rather than mRNA expression levels.

**Results:**

In this paper, we propose a computational approach that integrates microarray expression data with the transcription factor binding site information to systematically identify transcription factors associated with patient survival given a specific cancer type. This approach was applied to two gene expression data sets for breast cancer and acute myeloid leukemia. We found that two transcription factor families, the steroid nuclear receptor family and the ATF/CREB family, are significantly correlated with the survival of patients with breast cancer; and that a transcription factor named T-cell acute lymphocytic leukemia 1 is significantly correlated with acute myeloid leukemia patient survival.

**Conclusion:**

Our analysis identifies transcription factors associating with patient survival and provides insight into the regulatory mechanism underlying the breast cancer and leukemia. The transcription factors identified by our method are biologically meaningful and consistent with prior knowledge. As an insightful tool, this approach can also be applied to other microarray cancer data sets to help researchers better understand the intricate relationship between transcription factors and diseases.

## Background

Transcription factors (TFs) play important roles in the regulation of many biological processes, such as cell proliferation, cell cycle progression, and apoptosis [1,2]. Aberrant expression or activation/inactivation of TFs has been implicated in a variety of human cancer types [3-6]. As a matter of fact, a large number of oncogenes and tumor suppressor genes are actually TFs in nature [7]. P53, the most well studied tumor suppressor gene, has been found to mutate in over 50% of human cancers, mostly impairing its capability of transcriptional activation [8].

Association between TF expression and patient survival has been demonstrated in various cancer types [9-15]. Bamham et al. showed that in patients with diffuse large B-cell lymphoma (DLBCL) the transcription factor FOXP1-positive group had a significant decreased overall survival in comparison with the FOXP1-negative group (P = 0.0001) [12]. Anttilla et al. found that the expression level of cytoplasmic AP-2alpha, a transcription factor, is positively correlated with patient survival in epithelial ovarian cancer [15]. In lung adenocarcinoma, positive thyroid transcription factor 1 (TTF1) staining is strongly correlated with the survival of patients [11]. In gastric cancer, expression of the transcription factor Sp1 is negatively correlated with patient survival [13]. These studies indicate the importance of TFs in cancers as well as their prognostic value in clinical outcome predictions. Nevertheless, systematic association between TF activities (the capability for a TF to regulate gene expression) and patient survival has not previously been investigated due to the lack of high-throughput techniques to measure TF activities.

In cancer research, microarray technologies have been widely used to identify differentially expressed genes [16], to classify tumor samples into different sub-types [17], to predict clinical outcome based on gene expression profiles and so on [18]. However, in general, gene expression profiles in microarray data represent the down-stream readout of a few genetic alterations such as mutations, amplifications and deletions [19]. The regulatory mechanisms underlying the observed expression changes (e.g. the alterations in TF activities) are often not directly observable from the microarray data due to relatively low abundance of TF mRNAs and post-transcriptional modifications to TFs. Namely, the mRNA expression levels for TFs may not reflect their protein abundance or transcription regulatory activities. As a consequence, a mutation in the P53 gene, for instance, may not be reflected by its own expression change, but we would more likely observe the differential expression of its target genes. Thus, it is useful to infer the activity alterations of TFs in cancers from the expression changes of their target genes.

For many microarray cancer data sets, the survival information of patients after diagnosis is also provided. With this kind of data at hand, we propose a method to infer TF activities and identify TFs that are associated with patient survival in a systematic manner. Given gene expression profiles for tumor samples, we use the BASE method [20,21] to infer TF activities based on expression changes of their target genes. The complete list of target genes for human TFs is generally not available, so we used computational methods to predict the TF-gene regulatory relationships by examining the occurrence of TF binding sites (represented as positional weighted matrices, PWMs) within the promoter-proximal regions of genes. The resulting TF-gene binding affinity profiles were taken together with gene expression profiles as inputs to the BASE algorithm to infer the activities of TFs (PWMs) in each patient sample. We obtained 565 PWMs from the TRANSFAC database [22] and inferred their activities (reflect the activities of TFs binding with them) in each sample of the given microarray cancer data. We then identified all the PWMs whose activities were significantly correlated with patient survival.

We applied our method to two microarray data sets, a breast cancer data set with ER-positive and ER-negative subtypes [18] and a leukemia data set [23]. In breast cancer, the activities of steroid nuclear receptors and the ATF/CREB family are significantly correlated with the disease-free survival time of patients. In leukemia, TAL1 (T-cell acute lymphocytic leukemia 1) activity is significantly correlated with patient survival. Further investigation of these TFs may provide new insight into the mechanisms of tumorigenesis in breast cancer and leukemia. Moreover, our method can be readily applied to other microarray cancer data sets.

## Results and discussion

### Overview of breast cancer analysis

565 PWMs were obtained from the TRANSFAC database [22], and for each of them a binding potential profile was created by investigating its presence in the upstream promoter region of all human genes. These binding profiles were integrated with gene expression profiles for 98 breast cancer samples [18] to infer TF activities. For each of these 565 PWMs, our calculations yielded an activity profile, which represents the relative activities of the TF associated with the PWM in these samples. The correlations between these PWM activity profiles and the patient survival times were calculated to identify the PWMs (TFs) that are associated with patient survival. In total, we identified 26 PWMs at the 0.01 significance level (Q < 0.01); 6 of these are positively associated with patient survival while 20 are negatively associated, as shown in Table 1. We define a PWM as a positive predictor when its inferred activity is positively correlated with patient survival. Conversely, a PWM is called a negative predictor when its inferred activity is negatively correlated with patient survival. We note that the survival times for breast cancer patients in this dataset are actually represented as disease-free survival time (referred as "survival time" in this paper for simplification), denoted as the time from first diagnosis of breast cancer to development of distant metastases.

**Table 1:** PWMs associated with patient survival in breast cancer.

**PWM**	**TF**	**Correlation**	**q-value**	**PWM**	**TF**	**Correlation**	**q-value**
V$PR_02	PR	-0.46	0.00096	V$GRE_C	GR	-0.37	0.0051
V$E2F_03	E2F	-0.43	0.0012	V$SRF_Q5_01	SRF	-0.36	0.0060
V$CREBP1_Q2	CRE-BP1	-0.44	0.0013	V$HELIOSA_02	Helios	-0.35	0.0077
V$AR_02	AR	-0.41	0.0017	V$CREBATF_Q6	CREBATF	-0.35	0.0078
V$GR_01	GR	-0.40	0.0017	V$AR_01	AR	-0.35	0.0079
V$PAX3_B	PAX3	-0.41	0.0019	V$TAACC_B	Lentiviral	-0.35	0.0080
V$PR_01	PR	-0.41	0.0019	V$NFY_01	NF-Y	-0.34	0.0095
V$CREB_Q4	CREB	-0.40	0.0022	V$LXR_DR4_Q3	LXR	0.37	0.0090
V$AR_03	AR	-0.38	0.0033	V$S8_01	S8	0.39	0.0049
V$ATF4_Q2	ATF4	-0.39	0.0033	V$PAX9_B	PAX9	0.44	0.0015
V$OCT1_B	OCT1	-0.38	0.0037	V$MAF_Q6	MAF	0.40	0.0038
V$CHX10_01	CHX10	-0.37	0.0046	V$CEBPDELTA_Q6	C/EBPdelta	0.39	0.0055
V$CEBP_C	C/EBP	-0.37	0.0047	V$HNF3_Q6	HNF-3	0.40	0.0056

The table includes 20 negative and 6 positive PWM predictors that are significantly associated with patient survival at the 1% FDR. Correlation is based on the Spearman correlation coefficient between PWM activity and patient survival.

### Negative PWM predictors for patient survival

**Figure 1:**
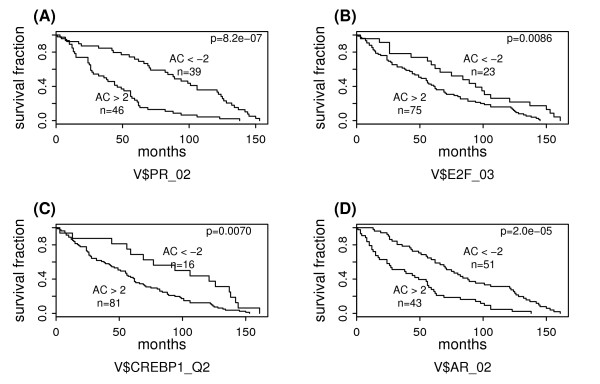
**Survival analysis of breast cancer subgroups defined based on activities of negative PWM predictors**. The "AC>2" and "AC<-2" subgroups are defined based on the AC scores of V$PR_02 in (A), V$E2F_03 in (B), V$CREBP1_Q2 in (C), or V$AR_02 in (D). The survival curves are estimated using the Kaplan-Meier method and the difference between subgroups is examined by the log-rank test.

From the 20 negative PWM predictors, 7 are binding motifs for the steroid nuclear receptor TF family: 2 for progesterone receptor (PR), 3 for androgen receptor (AR), and 2 for glucocorticoid receptor (GR). For example, the activities of V$PR_02 (PR binding motif) are negatively associated with patient survival (r = -0.46, Q = 0.00096). Based on the inferred activities of V$PR_02, we define two groups of cancer samples: group I (AC scores < -2) and group II (AC scores > 2). Figure 1A shows their survival curves obtained by the Kaplan-Meier method. The log-rank test indicates that patients in group I have significantly longer survival times than those in group II (P = 8.2E-7). Similarly, the negative association of V$AR_02 with patient survival (AR binding motif) is suggested by their correlation, -0.41 (Q = 0.0017), as well as the survival curves shown in Figure 1D. We use ± 2 as the cut-off values because an AC score within [-2,2] suggests no significant activity change of the corresponding PWM (TF) in the sample relative to the common reference. We have repeated the analysis using other cut-off values ranging from ± 3 to ± 6 and similar results were obtained.

These results are strongly supported by previous studies of association between steroid nuclear receptors and breast cancer. As a subfamily of the nuclear receptor TF superfamily, steroid nuclear receptors bind specifically to steroid hormones (e.g. androgen and estrogen) and mediate transcriptional regulation. Their involvement in growth, development and progression of breast cancer has been well established. First, ER, PR, AR and GR have been found to be frequently co-expressed in breast tumors; specifically, ER and PR are expressed in 70–80% and 70–90% of primary breast tumors, respectively [24], AR is expressed in 70–90% of primary breast tumors and 75% of breast cancer metastases [24], and GR is expressed in over 50% of human breast specimen [25]. Second, mutations or functional polymorphisms in steroid nuclear receptors cause or are associated with breast cancer [26-30]; for example, a germline mutation in the AR gene was reported as the causation of breast cancer in two brothers [31]. Third, therapeutic strategies directed at inhibiting activities of steroid nuclear hormones have been widely used for the treatment of breast cancer, e.g. the tamoxifen therapy for ER-positive breast cancer [32]. Fourth, ER, PR, AR and GR have been used as standard biomarkers of breast cancer. According to the status of these receptors, breast cancer has been categorized into different sub-types, e.g. ER-positive/PR-negative breast cancer. For different subtypes, different therapeutic treatments should be applied. The clinical outcome (the response to a certain therapeutic treatment) can be predicted based on the activities of these receptors [33,34]. Our results indicate that the activities of PWMs for PR, AR and GR are significantly correlated with survival times of breast cancer patients. As known, certain therapeutic treatments such as hormone therapy may lead to expression or activity change of steroid nuclear receptors. In our case, however, this possibility can be ruled out, since all patients in our analysis were treated by modified radical mastectomy or breast conserving treatment and no hormone therapy was applied [18]. Therefore, the association between these TFs with patient survival is not caused by the treatment effect.

Another TF family related to the negative PWM predictors is the ATF/CREB family. Among the 20 negative PWM predictors, 4 are binding motifs of the TFs in this family: V$CREBP1_Q2, V$CREB_Q4, V$ATF4_Q2 and V$CREBATF_Q6, which correspond to CRE-BP1 (ATF2), CREB (cAMP response-element binding protein), ATF4, and CREBATF, respectively. For instance, the correlation between the activity profile of V$CREBP1_Q2 and the patient survival is -0.44 (Q = 0.0013). When we define two patient groups based on the inferred AC scores of V$CREBP1_Q2, the low AC score group has significantly longer survival times than the high AC score group, as shown in Figure 1C.

The ATF/CREB family represents a large group of basic-region leucine zipper (bZIP) TFs, which have diverse functions in controlling cell proliferation and apoptosis [35]. In those ATF/CREB family members identified by our analysis, CRE-BP1/ATF2 has been implicated in transcriptional control of stress response genes [36]; CREB is involved in modulating transcription in response to intracellular cAMP concentrations [37] and ATF4 acts as negative regulator of cAMP responsive element (CRE)- dependent transcription [38]. Direct correlation between ATF/CREB family and breast cancer has never been reported, but several recent studies raise the possibility of its regulatory roles in human breast cancer. (1) They may act as co-activators for nuclear receptors, which are well-established risk factors of breast cancer, as mentioned above. For instance, CREB has been shown to be a co-activator of AR and mediates cross-talk with AP-1 [39]. (2) The ATF-2 mRNA levels in human breast cancers were lower than those in normal breast tissues [40]. (3) Studies in mouse models have shown that Atf2+/- mice were highly prone to mammary tumors and that ATF2 may act as a tumor susceptibility gene of mammary tumors [40,41]. (4) Transcriptional regulation of mouse brac2 gene, which together with brac1 is responsible for most hereditary breast cancers, has been shown to be driven by this TF family [42]. Consistently, our results indicate that the activities of TFs in the ATF/CREB family are negatively correlated with survival times of breast cancer patients.

In addition to the PWMs for TFs in the steroid nuclear receptor and the ATF/CREB families, there are several other negative PWM predictors for patient survival in breast cancer. For example, the AC scores of the E2F binding motif V$E2F_03 are associated with patient survival as revealed by their negative correlation -0.43 (Q = 0.0012). The predictability of V$E2F_03 to patient survival is also revealed by the survival curves of the two patient groups based on its activities as shown in Figure 1B. E2F plays a key role in the mammalian cell cycle regulation and many of its target genes have a function in cellular proliferation [2]. High activity of PWM for E2F may be indicative of high proliferation rate of cells. Furthermore, the involvement of E2F in breast cancer has been demonstrated in several studies [43]. Taking these together, it is not surprising to observe the negative correlation between the activity of PWM for E2F and the survival times of breast cancer patients.

### Positive PWM predictors for patient survival

**Figure 2:**
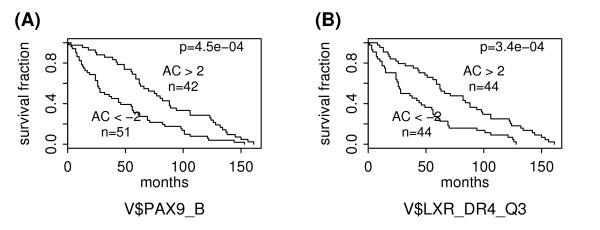
**Survival analysis of breast cancer subgroups defined based on activities of positive PWM predictors**. The "AC>2" and "AC<-2" subgroups are defined based on the AC scores of V$PAX9_B in (A) or V$LXR_DR4_Q3 in (B).

As shown in Table 1, there are 6 positive PWM predictors for patient survival in breast cancer. Figure 2 shows the ability of V$PAX9_B and V$LXR_DR4_Q3 to predict patient survival times. As shown, patients in the group with higher AC scores of V$PAX9_B (Figure 2A) or V$LXR_DR4_Q3 (Figure 2B) have significantly longer survival times than those in the group with lower AC scores. The p-values are 4.5E-4 and 3.4E-4 for V$PAX9_B and V$LXR_DR4_Q3, respectively. LXR, the so-called liver X receptor, also belongs to the superfamily of nuclear receptors but is not a member of the steroid nuclear receptor sub-family. LXR controls estrogen homeostasis by regulating the hepatic expression of estrogen sulfotransferase (Est), an enzyme critical for metabolic estrogen deactivation [44]. Moreover, genetic or pharmacological activation of LXR results in Est induction, which in turn inhibits breast cancer growth in a nude mouse model of tumorigenicity [44]. The complete results for positive and negative PWM predictors for patient survival can be found in the Additional file 1.

We identified the PWM activity profiles that can best predict patient survival based on the Cox proportional-hazards model [45,46]. The model results in 20 significant PWMs, among which 9 have a positive effect (V$PAX9_B, V$ISRE_01, V$LXR_DR4_Q3, V$AHR_Q5, V$USF_01, V$S8_01, V$LEF1_Q2_01, V$NRSF_01, V$MEF2_01) and 11 have a negative effect (V$AR_02, V$SRF_Q5_01, V$E2F1_Q3, V$CREBP1_Q2, V$EVI1_06, V$E2F_03, V$PAX3_B, V$MYCMAX_B, V$CHX10_01, V$E2F_Q2, V$CREBATF_Q6) on patient survival. To investigate the effect of sample size, we randomly selected a fraction of the 98 samples (70–95%) and applied our analysis to these subsets. As expected, more significant PWMs were identified when more samples were used owning to the increase of statistical power. Meanwhile, similar results have been obtained for different randomly selected subsets. Moreover, we have applied our analysis to another breast cancer data set performed by van de Vijver et al [47] (some samples in this data have also been used in van't Veer's study [18]; these samples were excluded from our analysis) and the results again highlighted the critical roles of the nuclear receptor and ATF/CREB TF families in breast cancer. Among the 39 PWMs that are significantly correlated with patient survival (Q < 10%), 5 are binding motifs for nuclear receptors (3 for AR, 1 for PR and 1 for GR) and 7 others for ATF/CRBP TF family. However, there are only 15 PWMs in common between the two datasets when using a FDR of 10% (39 PWMs for van de Vijder's data and 77 for van't Veer's data). Though significant (P = 1E-4), the overlap is not large, which may reflect the sample difference between the two data sets. While all patients in van't Veer data had lymph node-negative breast cancer, approximately half of the patients in van de Vijver data were lymph node-positive.

The method we suggest is intrinsically less sensitive to the platform effect, since it measures an average involvement of TFs. To investigate the platform effect, we applied our method to the breast cancer data set by Wang et al. [48], which contained the expression profiles for 286 samples measured by the Affymetrix one-channel arrays (in our analysis we only used the expression profiles for non-censored samples). The results were consistent with those from the cDNA array platforms (the van't Veer's and the van de Vijder's data). At the 0.01 false discovery rate, we identified a total of 9 significant PWMs including V$AR_03 (ρ = -0.36, Q = 0.008) and V$CREB_02 (ρ = -0.35, Q = 0.01). If we relax the false discovery rate to 0.1, 2 PWMs for AR and 11 PWMs for the ATF/CREB family are detected to be negative predictors for patient survival.

### Logistic regression model for patient survival prediction

A logistic regression model was created to predict the prognostic outcome of breast cancer patient survival based on the activity inferences of only 4 PWMs: V$PR_02, V$E2F_03, V$CREBP1_Q2, and V$PAX9_B. In the model, the inferred AC scores of these PWMs are used as predictors. The 98 patients are divided into two categories according to their survival times. Patients that did not relapse for at least 60 months are included in the good prognosis category; the remaining patients are included in the poor prognosis category. The predictive power of this model was assessed using the leave-one-out cross-validation method. Our results indicate that this model correctly predicted the actual outcome for 75 out of 98 patients (76%), with 11 poor prognosis and 12 good prognosis patients assigned to the opposite category. The logistic regression model based on gene expression levels instead achieves a prediction accuracy rate of 83% [18], however, as many as 70 well established marker genes are included in this model. Despite the small decrease in prediction accuracy, our results indicate that it might be useful in practice to include TF activity information for breast cancer prognosis. We would like to highlight our belief that the small loss of accuracy is overshadowed by the biological gain in the understanding of this cancer, since, in addition to the predictions, our analysis provides a list of candidate transcription factors that may be involved in the cancer mechanism.

### Activity versus expression level of TFs

As demonstrated above the activity score of TFs has a strong correlation and predictive power towards patient survival, however, a TF's expression level from the microarray experiments, is generally either less correlated or not at all correlated to patient survival. For instance, the correlation of patient survival with ATF4 mRNA expression (ρ = -0.24, Q = 0.052) is much less than its correlation with the inferred activity for V$ATF4_Q2 (ρ = -0.39, Q = 0.0033). Biological functions mediated by TFs are largely determined by their activities rather than expression levels, hence it is more reasonable and sensitive to examine the correlation between TF activity and patient survival.

We also calculated the correlations between the expression levels of steroid nuclear receptors and patient survival. Interestingly, we find that ER, PR, AR and GR are positively correlated with patient survival at the expression level, with the Spearman correlation coefficients 0.45 (Q = 0.0035), 0.34 (Q = 0.053), 0.33 (Q = 0.059) and 0.04 (Q = 0.98), respectively. In contrast, as described above, the inferred AC scores of the PWMs for PR, AR, and GR are negatively correlated with patient survival. We compared the expression levels as well as AC scores of AR in ER-positive (n = 53) and ER-negative (n = 44) breast tumors using the Wilcoxon rank sum test. We find that the expression levels of AR in the ER-positive group are significantly higher than those in the ER-negative group (p-value = 8.8E-6), whereas the AC scores of AR binding motif (V$AR_02) in the ER-positive group are significantly lower than those in the ER-negative group (P = 1.1E-6). This indicates that PWMs for PR, AR and GR may predominantly mediate transcriptional repression of these TFs, because a higher AC score indicates higher activity of transcriptional activators but a lower activity of transcriptional repressors. Alternatively, it may also result from the difference between expression level and activity/protein level of these receptors, which are caused by post-transcriptional modifications, interactions with other co-activators/co-repressors, or other complications. The difference between expression level and protein/activity level has been frequently observed. For example, dihydrotestosterone treatment for MDA-453, a breast cancer cell line, has been found to decrease total AR mRNA but increase AR protein [49]. On the other hand, we should note that our method may fail to identify some cancer related TFs. For example, we do not find out the correlation between ER and patient survival based on the inferred activities for ER PWMs. The possible reasons are: (1) the PWMs for ER are not in high quality and do not reflect their binding preference correctly; (2) ER regulates gene expression by distant binding sites and focus on core promoter regions fails to reveal the true TF-gene relationships. A recent ChIP-chip experiment indicates that only 4% of ER binding sites can be mapped to 1-kb promoter-proximal regions [50].

ER status is a significant risk factor for breast cancer. As shown by previous studies, our results show that patients in the ER-positive breast cancer group have significantly longer survival times than those in the ER-negative group (P = 1.5E-4 according to the log rank test, see the figure in the Additional file 2). Therefore, in the following analysis, we divided patients into ER-positive and ER-negative breast cancer groups and identified PWMs associated with survival times in these two groups separately.

### Significant PWMs in ER-positive breast cancer

**Figure 3:**
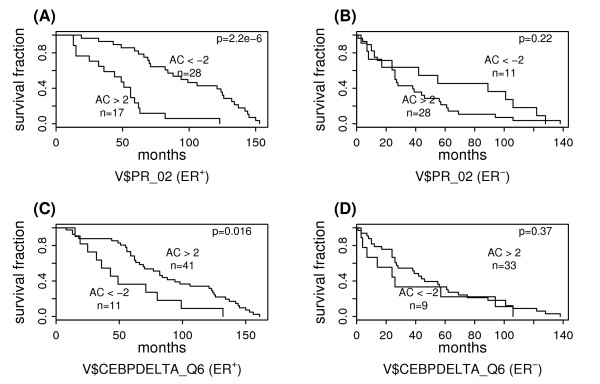
**Survival analysis of subgroups defined based on PWM activities in ER-positive and ER-negative breast cancers**. The "AC>2" and "AC<-2" subgroups are defined based on the AC scores of (A): V$PR_02 in ER-positive breast cancer, (B): V$PR_02 in ER-negative breast cancer, (C): V$CEBPDELTA_Q6 in ER-positive breast cancer, and (D): V$CEBPDELTA_Q6 in ER-negative breast cancer.

Table 2 shows the PWMs that are associated with the survival of ER-positive breast cancer patients; 6 are negative predictors and 1 is a positive predictor. Among the negative PWM predictors, 2 correspond to PR and the others correspond to GR, CRE-BP1/ATF2, NF-Y, and DEAF1. In ER-negative breast cancers, however, none of them is associated with patient survival at the 0.10 significance level. Figure 3A shows the survival curves of two sub-groups of ER-positive breast cancer patients. As shown, based on V$PR_02, the low AC score sub-group survives significantly longer than the high AC score sub-group (P = 2.2E-6). In ER-negative breast cancer patients, however, the activity of V$PR_02 provides no predictive power regarding the patient survival time as shown in Figure 3B. V$CEBPDELTA_Q6 is the only positive PWM predictor in ER-positive breast cancer. Its predictive power in ER-positive and ER-negative breast cancers is shown in Figure 3C and Figure 3D, respectively. The complete results for significant PWM predictors in ER-positive breast cancer can be found in the Additional file 3.

**Table 2:** PWMs associated with patient survival in ER-positive breast cancer.

		**ER+ Breast Cancer**		**ER- Breast Cancer**	
**PWM**	**TF**	**Correlation**	**q-value**	**Correlation**	**q-value**
V$PR_02	PR	-0.53	0.016	-0.16	0.81
V$CEBPDELTA_Q6	C/EBPdelta	0.49	0.057	0.16	0.98
V$CREBP1_Q2	CRE-BP1	-0.43	0.079	-0.44	0.15
V$PR_01	PR	-0.44	0.083	-0.14	0.82
V$GR_01	GR	-0.43	0.086	-0.29	0.47
V$NFY_01	NF-Y	-0.46	0.094	-0.24	0.55
V$DEAF1_01	DEAF1	-0.44	0.096	-0.06	0.91

For each PWM, the Spearman correlation coefficients between PWM activity and patient survival in both ER-positive (ER^+^) and ER-negative (ER^-^) breast cancer are shown.

### Significant PWMs in ER-negative breast cancer

**Figure 4:**
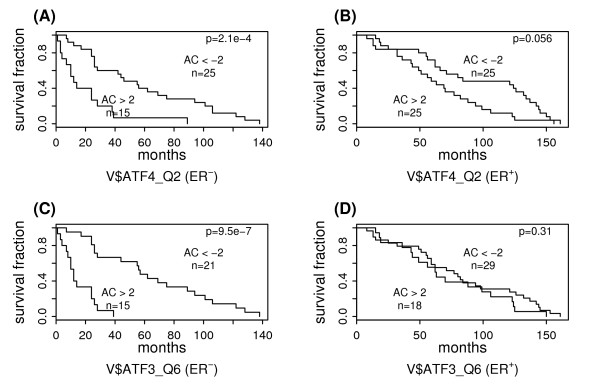
**Survival analysis of subgroups defined based on PWM activities in ER-negative and ER-positive breast cancers**. The "AC>2" and "AC<-2" subgroups are defined based on the AC scores of (A): V$ATF4_Q2 in ER-negative breast cancer, (B): V$ATF4_Q2 in ER-positive breast cancer, (C): V$ATF3_Q6 in ER-negative breast cancer, and (D): V$ATF3_Q6 in ER-positive breast cancer.

Table 3 shows 3 PWMs associated with the survival of ER-negative breast cancer patients; all of which are negative predictors. They are respectively binding motifs for ATF4, CREB and ATF3, all belonging to the ATF/CREB family. In fact, among the top 9 PWMs which are most correlated with the survival of ER-negative breast cancer patients, 7 are binding motifs of the TFs in ATF/CREB family. The survival predictive power of V$ATF4_Q2 and V$ATF3_Q6 in ER-negative and ER-positive breast cancers is shown in Figure 4A–D. In ER-negative breast cancer the low AC score sub-groups have significant longer survival times than the high AC score sub-group. But in ER-positive breast cancers, no significant difference in the survival time between these two sub-groups is observed at the 0.05 significance level. The complete results for significant PWM predictors in ER-negative breast cancer can be found in the Additional file 4.

**Table 3:** PWMs associated with patient survival in ER-negative breast cancer.

		**ER+ Breast Cancer**		**ER- Breast Cancer**	
**PWM**	**TF**	**Correlation**	**q-value**	**Correlation**	**q-value**
V$ATF4_Q2	ATF4	-0.34	0.28	-0.60	0.0080
V$CREB_Q4	CREB	-0.21	0.69	-0.54	0.030
V$ATF3_Q6	ATF3	-0.18	0.76	-0.49	0.078

For each PWM, the Spearman correlation coefficients between PWM activity and patient survival in both ER-positive (ER^+^) and ER-negative (ER^-^) breast cancer are shown.

In addition to van't Veer's data [18], we have also applied our analysis to several other breast cancer data sets [48,51-54] and for two of them the transcription factors associated with patient survival were successfully identified (FDR < 0.01). Both data sets supported the involvement of the steroid nuclear receptors and the ATF/CREB TF family members in breast cancer. But it should be noted that the specific association of the ATF/CREB family with ER-negative breast cancer was detected only in van't Veer's data. The discordance may reflect the difference in sample preparation and patient treatment. Particularly, it is known that the van't Veer's data may include a very biased selection of patients and this bias explains at least partly the fact that approximately 10% of genes show a significant association with survival in van't Veer's data, while in other data sets the proportion is only about 1% [55]. Therefore, the specific association of the steroid nuclear receptors with ER-positive and the ATF/CREB family with ER-negative breast cancer may result from this bias and should be subject to more careful investigation in future studies.

### Summary of breast cancer analysis

Our results indicate that the steroid nuclear receptor and the ATF/CREB families are associated with the survival breast cancer patients. In van't Veer's data set we found that the steroid nuclear receptor family is associated with the ER-positive breast cancer, whereas the ATF/CREB family is associated with the ER-negative breast cancer patients. The involvement of steroid nuclear receptors in ER-positive breast cancers has been known for decades, but the functions of ATF/CREB family in ER-negative breast cancers are largely unknown. Further investigation of this TF family may shed new light on the transcriptional regulation in breast cancers, especially in the ER-negative breast cancers. For ER-positive breast cancers, hormone therapy that target to steroid nuclear receptors has achieved great success. For example, tamoxifen blocks estrogen's ability to trigger abnormal cell growth, and has been used to treat or prevent ER-positive breast cancers. But for ER-negative breast cancers, none of these drugs targeting steroid nuclear receptors is effective. The specific association of the ATF/CREB family with ER-negative breast cancer revealed in van't Veer's data deserves further experimental validation and transcription factors in this family may serve as the targets of new drugs designed to treat ER-negative breast cancers.

Several studies have been performed to explore the transcriptional regulatory programs underlying distinct breast cancer phenotypes such as estrogen receptor status and histological grades [56-59]. All these studies apply a similar strategy: to identify a set of genes that are differentially expressed between two breast cancer categories (e.g. ER+ versus ER-) and then investigate the enrichment of motifs in these genes. For example, Niida et al. searched for cis-regulatory motifs associated with given histological grades and prognosis, and found that motifs bound by ELK1, E2F, NRF1 and NFY are potential regulatory motifs that positively correlate with malignant progression of breast cancer [57]. In contrast, our method applies a quite different strategy to identify PWMs associated with breast cancer patient survival. We infer the transcriptional activity profiles of all PWMs across the samples and then identify significant PWMs by examining the correlation of their activity profiles with patient survival. Despite the difference in methodology, our analysis confirms some of previous findings: e.g. we also detect the correlation of E2F and NFY with breast cancer prognosis as revealed by Nidda et al [57]. A collective application of these approaches should be useful and can provide insight into the disease mechanism for other cancer types.

Instead of BASE, the activity profiles for PWMs can also be inferred by using other methods such as the REDUCE [60], network component analysis [61], MA-Networker algorithm [62], and partial least squares regression method [63]. These methods are based on a model assuming a linear relationship between gene expression changes and TF-gene binding affinities. The linear models provide a simultaneous inference of all TF activities in the model and thereby take the overlapping of TF target gene sets into account; whereas the BASE algorithm considers each TF independently. When BASE is used for TF activity inference, we would expect to obtain a more complete list of TFs that are associated with patient survival. In contrast, the linear model based methods would result in a set of TFs that can best predict patient survival if combined with Cox proportional-hazards model.

### Acute myeloid leukemia

In the leukemia data, cDNA microarrays were used to measure gene expression levels in peripheral-blood or bone marrow samples from 116 patients with acute myeloid leukemia (AML) [23]. The survival times after diagnosis of these patients were also available. We applied our analysis to this data set to identify the TFs (PWMs) associated with the survival of AML patients.

**Figure 5:**
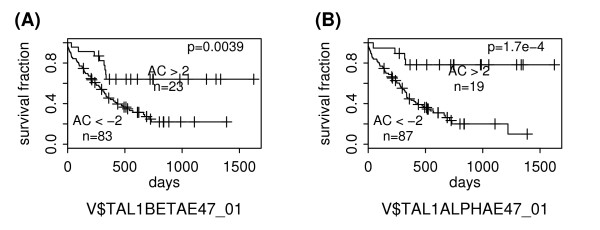
**Survival analysis of AML subgroups defined based on PWM activities**. The "AC>2" and "AC<-2" subgroups are defined based on the AC scores of V$TAL1BETAE47_01 in (A) or V$TAL1ALPHAE47_01 in (B). The "+" signs mark the events at which a sample is censored.

We identified two PWMs at the 0.10 significance level (Q < 0.10): V$TAL1BETAE47_01 and V$TAL1ALPHAE47_01. They are similar in sequence and correspond to the transcription factors TAL1-alpha and TAL1-beta, respectively. Their ability to predict AML patient survival is shown in Figure 5. It is notable that the majority of AML patients have an AC score less than -2 (83 out of 116 for V$TAL1BETAE47_01 and 87 out of 116 for V$TAL1BETAE47_01), suggesting the enhanced activities of TAL1-beta and TAL1-alpha (function as transcriptional repressors) in AML samples.

TAL1, the so-called T-cell acute lymphocytic leukemia 1, is a member of the basic HLH family of transcription factors and is involved in the regulation of hematopoiesis [64,65]. The TAL1 gene encodes two polypeptides, full-length TAL1α and N-terminally truncated polypeptide TAL1β [66]. Aberrant activation of TAL1 in the T-cell lineage by recurrent chromosomal translocation, chromosomal deletion, and other unknown mechanisms is implicated as the major pathway for the development of T-cell acute lymphoblastic leukemia (T-ALL) [67,68]. According to the prevailing model of TAL1-induced leukemogenesis, TAL1 acts as a transcriptional repressor through heterodimerization with the transcription factors E2A and HEB, leading to the block of their transcriptional activities [69,70]. Although most studies regarding TAL1 are focused on its association with T-ALL, our results indicate that it may also be critical in the development of AML. Further investigation of TAL1 function in AML patients may enable us to better understand the underlying mechanisms of oncogenesis, as well as to identify the appropriate therapeutic strategies for AML. The complete results for significant PWM predictors in acute myeloid leukemia can be found in the Additional file 5.

## Conclusion

In this paper, we propose a computational approach to systematically identify TFs (PWMs) associated with patient survival in human cancer. This approach was applied to the breast cancer and AML microarray expression data sets. In breast cancer, we find that the members of two TF families, the steroid nuclear receptor and the ATF/CREB families, are significantly associated with patient survival. This method can also be used to identify transcription factors associated with a specific cancer subtype. For example, we find that in van't Veer's breast cancer data set the steroid receptor and the ATF/CREB families are respectively associated with patient survival in ER-positive and ER-negative breast cancer. Our analysis reveals the possible regulatory programs underlying different breast cancer subtypes, which are largely unknown and deserve further studies. The involvement of the transcription factor TAL1 in T-ALL has been well established; however, our results indicate that TAL1 may also play critical roles in AML. Our approach provides a useful tool to investigate TFs associated with patient survival and is ready to be used for other microarray cancer data sets.

## Methods

### Overview

In this paper, we aimed to identify transcription factors (TFs) associated with cancer patient survival by integrating gene expression data, survival data, and transcription factor binding site (TFBS) information. First, we used a method called BASE [20,21] to infer the TF activities from cancer microarray data. Second, we downloaded 565 positional weighted matrices (PWM) from the TRANSFAC database, which represent the TFBSs for 365 TFs in vertebrates. Based on these PWMs, we inferred the TF activities in all tumor samples, resulting in 565 PWM activity profiles. Third, the correlations between these activity profiles and patient survival were calculated and their significances were assessed using permutation testing. Finally, to show the advantage of TF activities in terms of patient survival prediction, we compared the prediction results of the linear regression models based on TF activities with those based on gene expressions.

### Cancer expression data sets

Two cancer expression and survival data sets were involved in the analysis of this paper: a breast cancer data set and a leukemia data set. For the breast cancer data, expressions of approximately 25,000 human genes were measured using the cDNA arrays for tumor samples collected from 98 patients with primary invasive breast carcinoma [18]. In addition to the expression data, the survival data as well as the histological data for these 98 patients are also available [71]. The survival data is represented as the disease-free survival times for all 98 patients with no missing values (i.e. no sample has been censored).

To obtain the leukemia data, 65 peripheral blood samples and 54 marrow samples from 116 patients with acute myeloid leukemia (AML) were collected [23]. For each sample, the expression levels of 26,260 human genes were measured using the cDNA array. The complete microarray data set is available at the gene expression omnibus (GEO) with accession number GSE425. In addition, the survival data for these 116 patients is also available [23].

### Positional weighted matrices for human TFs

From the TRANSFAC database, we downloaded 565 PWMs for 365 vertebrate TFs. The MATCH program was used to examine the presence of each of these PWMs in the upstream regions (from the transcription start site to 1000 bp upstream) of all human genes, which is available from the UCSC Genome Browser [72]. The pre-calculated cut-offs for these PWMs (provided by the MATCH program) were used to minimize the false positive rate [73]. For each PWM, the MATCH program outputs all the potential binding sites as well as their matching-scores in the upstream regions of all genes. Based on these outputs, we constructed a binding score matrix [B_ij_] of size N × M, where N and M are the numbers of genes (N = 20375) and PWMs (M = 565), respectively. B_ij _was calculated by aggregating the matching-scores of all the binding sites of PWM *j *in the upstream region of gene *i*. The score was set to 0 when no binding site was found in the upstream region of a gene. This binding score matrix B reflects the binding potential of PWMs to genes.

### Transcriptional activity inference

BASE was the method used in this study to infer the relationship between TFs and the survival probability of patients with different cancer types. This method makes use of gene expression data and TF-gene binding affinities. The intuition behind this method is that if a TF is related to the survival outcome of a patient given a certain disease, then the genes that are regulated by this TF will be more differentially expressed between the patients that survive or die. This method is substantially different from finding a correlation, such as Pearson or Spearman correlation coefficients, between gene expression and binding affinity. We believe that a correlation coefficient should not be used in this study as the information that the BASE method is able to uncover is mainly contained at the extremities of the *e*_*i *_values (i.e. most differentially expressed genes); hence a correlation coefficient for the entire range of *e*_*i *_values would not be significant.

The BASE method can be described as follows; given a TF with binding affinity *b*_*i *_and an expression differentiation profile *e*_*i *_(i = 1,2,...,N and N is the number of genes), BASE calculates the correlation between these two profiles using a Kolmogorov-Smirnov test like method. First we sort *e*_*i *_in the decreasing order and reorder *b*_*i *_accordingly to obtain *e*_(*i*) _and *b*_(*i*)_, respectively. Then we calculate a function  and a reference function . *f*(*i*) can be regarded as the cumulative distribution function (CDF) for *e*_(*i*) _weighted by *b*_(*i*)_, and *g*_(*i*) _is the CDF for *e*_(*i*)_. If the TF is significantly activated, we would expect genes with higher binding affinities (b values) to have higher expression changes (e values), and thereby *f*(*i*) increases rapidly relative to *g*_(*i*)_. So we can use the maximum deviation between *f*(*i*) and *g*(*i*), denoted as pre-score ps*, to measure the correlation between profiles *e*_(*i*) _and *b*_(*i*)_. The significance of ps* is estimated by permutation test: permute *e*_*i *_and redo the above calculation to obtain the null distribution of pre-score. Finally, the pre-score ps* is normalized to obtain an activity change score (AC score), which can directly compared between different TFs. A more detailed description of BASE can be found in [20]. Essentially, the transcriptional inference is based on expression changes of the target genes of a TF. But the BASE method does not use a determined target gene set, instead, it utilizes the binding affinity profile that reflects the binding potential of a TF to genes. The BASE method is relatively robust to false predicted TF targets: since the activity change of a TF can be captured by AC score even if only a subset of its targets shows expression change. This is critical for PWM based analysis, as a considerable fraction of TFBSs from PWM searching is non-functional.

For each PWM, BASE calculates an AC score profile, indicating the relative activities of the corresponding TF in each of the tumor samples. A positive AC score indicates the activity enhancement for a transcriptional activator or the activity reduction for a transcriptional repressor; while a negative AC score indicates the opposite activity change.

### Identification of PWMs associated with patient survival

The above described transcriptional inference results in a total of 565 activity profiles, each corresponding to a PWM. We then calculated the Spearman correlation coefficients between each activity profile and the patient survival times, resulting in a correlation vector denoted as r. In order to estimate the significances of these correlations, we permuted the patient survival vector K (K = 10,000) times. Spearman correlation coefficients are recalculated between activity profiles and each of the permuted survival vectors, and denoted as the permutated correlation vector as π_k _for the k-th permutation, where k = 1, 2,...,K. We created a histogram of all these permutated correlations, and used this null distribution to compute the FDR q value for a given value r* in the original correlation vector r as following:



### Logistic regression model for patient survival prediction

A logistic regression model was constructed to predict the clinical outcome of patients with breast cancer. In this model, the predictors are the inferred activities of 4 PWMs that are most associated with patient survival. Two groups, the "good" and "poor" prognosis groups were defined based on the patient survival times. Out of the 98 patients, 46 who survived at least 60 months after diagnosis were categorized into the "good" prognosis group and the remaining 52 were categorized into the "poor" prognosis group. We used leave-one-out cross validation to evaluate the accuracy of this model. Specifically, each sample's prediction was obtained by the use of a model that was fit by the remaining 97 samples. This procedure was repeated until each sample was left out once, resulting in 98 predictions. We finally compared the predictions with the actual clinical outcome to estimate the prediction accuracy of our model.

### Cox proportional-hazards regression

We used the Cox proportional-hazards model to identify the PWM activity profiles that can best predict the patient survival. The shrinkage method, LASSO [74], was used for parameter estimation and the tuning parameter for LASSO was optimized by 10-fold cross validation. The R package "penalized" was implemented for above analysis.

## Authors' contributions

CC designed the method, wrote the code, carried out the analysis, and drafted the manuscript. LML and MG participated in design and coordination of the study. PA helped to analyze the data and draft the manuscript. All authors read and approved the final manuscript.

## Supplementary Material

Additional file 1 **Complete results for breast cancer**. Spearman correlation coefficient between PWM activity and survival is calculated based on 98 samples from patients with breast cancer. Click here for file

Additional file 2 **Survival analysis of ER-positive and ER-negative breast cancer subgroups**. The survival curves are estimated using the Kaplan-Meier method and the difference between subgroups is examined by the log-rank test. Click here for file

Additional file 3 **Complete results for ER-positive breast cancer**. Spearman correlation coefficient between PWM activity and survival is calculated based on 53 samples from patients with ER-positive breast cancer. Click here for file

Additional file 4 **Complete results for ER-negative breast cancer**. Spearman correlation coefficient between PWM activity and survival is calculated based on 44 samples from patients with ER-negative breast cancer. Click here for file

Additional file 5 **Complete results for acute myeloid leukemia**. Spearman correlation coefficient between PWM activity and survival is calculated based on 116 samples from patients with acute myeloid leukemia. Click here for file

## References

[B1] Hilger-Eversheim K, Moser M, Schorle H, Buettner R (2000). Regulatory roles of AP-2 transcription factors in vertebrate development, apoptosis and cell-cycle control. Gene.

[B2] Muller H, Helin K (2000). The E2F transcription factors: key regulators of cell proliferation. Biochim Biophys Acta.

[B3] Blancafort P, Chen EI, Gonzalez B, Bergquist S, Zijlstra A, Guthy D, Brachat A, Brakenhoff RH, Quigley JP, Erdmann D (2005). Genetic reprogramming of tumor cells by zinc finger transcription factors. Proc Natl Acad Sci USA.

[B4] Sakakura C, Hagiwara A, Miyagawa K, Nakashima S, Yoshikawa T, Kin S, Nakase Y, Ito K, Yamagishi H, Yazumi S (2005). Frequent downregulation of the runt domain transcription factors RUNX1, RUNX3 and their cofactor CBFB in gastric cancer. Int J Cancer.

[B5] Darnell JE (2002). Transcription factors as targets for cancer therapy. Nat Rev Cancer.

[B6] Gilliland DG (2001). The diverse role of the ETS family of transcription factors in cancer. Clin Cancer Res.

[B7] Introna M, Golay J (1999). How can oncogenic transcription factors cause cancer: a critical review of the myb story. Leukemia.

[B8] Strano S, Dell'Orso S, Di Agostino S, Fontemaggi G, Sacchi A, Blandino G (2007). Mutant p53: an oncogenic transcription factor. Oncogene.

[B9] Ma D, Nutt CL, Shanehsaz P, Peng X, Louis DN, Kaetzel DM (2005). Autocrine platelet-derived growth factor-dependent gene expression in glioblastoma cells is mediated largely by activation of the transcription factor sterol regulatory element binding protein and is associated with altered genotype and patient survival in human brain tumors. Cancer Res.

[B10] Berger AJ, Kluger HM, Li N, Kielhorn E, Halaban R, Ronai Z, Rimm DL (2003). Subcellular localization of activating transcription factor 2 in melanoma specimens predicts patient survival. Cancer Res.

[B11] Barlesi F, Pinot D, Legoffic A, Doddoli C, Chetaille B, Torre JP, Astoul P (2005). Positive thyroid transcription factor 1 staining strongly correlates with survival of patients with adenocarcinoma of the lung. Br J Cancer.

[B12] Banham AH, Connors JM, Brown PJ, Cordell JL, Ott G, Sreenivasan G, Farinha P, Horsman DE, Gascoyne RD (2005). Expression of the FOXP1 transcription factor is strongly associated with inferior survival in patients with diffuse large B-cell lymphoma. Clin Cancer Res.

[B13] Yao JC, Wang L, Wei D, Gong W, Hassan M, Wu TT, Mansfield P, Ajani J, Xie K (2004). Association between expression of transcription factor Sp1 and increased vascular endothelial growth factor expression, advanced stage, and poor survival in patients with resected gastric cancer. Clin Cancer Res.

[B14] Span PN, Manders P, Heuvel JJ, Thomas CM, Bosch RR, Beex LV, Sweep CG (2002). Expression of the transcription factor Ets-1 is an independent prognostic marker for relapse-free survival in breast cancer. Oncogene.

[B15] Anttila MA, Kellokoski JK, Moisio KI, Mitchell PJ, Saarikoski S, Syrjanen K, Kosma VM (2000). Expression of transcription factor AP-2alpha predicts survival in epithelial ovarian cancer. Br J Cancer.

[B16] Han H, Bearss DJ, Browne LW, Calaluce R, Nagle RB, Von Hoff DD (2002). Identification of differentially expressed genes in pancreatic cancer cells using cDNA microarray. Cancer Res.

[B17] Mischel PS, Cloughesy TF, Nelson SF (2004). DNA-microarray analysis of brain cancer: molecular classification for therapy. Nat Rev Neurosci.

[B18] van 't Veer LJ, Dai H, Vijver MJ van de, He YD, Hart AA, Mao M, Peterse HL, Kooy K van der, Marton MJ, Witteveen AT (2002). Gene expression profiling predicts clinical outcome of breast cancer. Nature.

[B19] Rhodes DR, Kalyana-Sundaram S, Mahavisno V, Barrette TR, Ghosh D, Chinnaiyan AM (2005). Mining for regulatory programs in the cancer transcriptome. Nat Genet.

[B20] Cheng C, Yan X, Sun F, Li LM (2007). Inferring activity changes of transcription factors by binding association with sorted expression profiles. BMC Bioinformatics.

[B21] Cheng C, Li LM (2008). Systematic identification of cell cycle regulated transcription factors from microarray time series data. BMC Genomics.

[B22] Matys V, Fricke E, Geffers R, Gossling E, Haubrock M, Hehl R, Hornischer K, Karas D, Kel AE, Kel-Margoulis OV (2003). TRANSFAC: transcriptional regulation, from patterns to profiles. Nucleic Acids Res.

[B23] Bullinger L, Dohner K, Bair E, Frohling S, Schlenk RF, Tibshirani R, Dohner H, Pollack JR (2004). Use of gene-expression profiling to identify prognostic subclasses in adult acute myeloid leukemia. N Engl J Med.

[B24] Birrell SN, Hall RE, Tilley WD (1998). Role of the androgen receptor in human breast cancer. J Mammary Gland Biol Neoplasia.

[B25] Allegra JC, Lippman ME, Thompson EB, Simon R, Barlock A, Green L, Huff KK, Do HM, Aitken SC, Warren R (1979). Relationship between the progesterone, androgen, and glucocorticoid receptor and response rate to endocrine therapy in metastatic breast cancer. Cancer Res.

[B26] Lillie EO, Bernstein L, Ursin G (2003). The role of androgens and polymorphisms in the androgen receptor in the epidemiology of breast cancer. Breast Cancer Res.

[B27] Spurdle AB, Antoniou AC, Duffy DL, Pandeya N, Kelemen L, Chen X, Peock S, Cook MR, Smith PL, Purdie DM (2005). The androgen receptor CAG repeat polymorphism and modification of breast cancer risk in BRCA1 and BRCA2 mutation carriers. Breast Cancer Res.

[B28] Curran JE, Lea RA, Rutherford S, Weinstein SR, Griffiths LR (2001). Association of estrogen receptor and glucocorticoid receptor gene polymorphisms with sporadic breast cancer. Int J Cancer.

[B29] De Vivo I, Hankinson SE, Colditz GA, Hunter DJ (2003). A functional polymorphism in the progesterone receptor gene is associated with an increase in breast cancer risk. Cancer Res.

[B30] Wang-Gohrke S, Chang-Claude J, Becher H, Kieback DG, Runnebaum IB (2000). Progesterone receptor gene polymorphism is associated with decreased risk for breast cancer by age 50. Cancer Res.

[B31] Wooster R, Mangion J, Eeles R, Smith S, Dowsett M, Averill D, Barrett-Lee P, Easton DF, Ponder BA, Stratton MR (1992). A germline mutation in the androgen receptor gene in two brothers with breast cancer and Reifenstein syndrome. Nat Genet.

[B32] Frasor J, Chang EC, Komm B, Lin CY, Vega VB, Liu ET, Miller LD, Smeds J, Bergh J, Katzenellenbogen BS (2006). Gene expression preferentially regulated by tamoxifen in breast cancer cells and correlations with clinical outcome. Cancer Res.

[B33] Buchanan G, Birrell SN, Peters AA, Bianco-Miotto T, Ramsay K, Cops EJ, Yang M, Harris JM, Simila HA, Moore NL (2005). Decreased androgen receptor levels and receptor function in breast cancer contribute to the failure of response to medroxyprogesterone acetate. Cancer Res.

[B34] Ma H, Bernstein L, Pike MC, Ursin G (2006). Reproductive factors and breast cancer risk according to joint estrogen and progesterone receptor status: a meta-analysis of epidemiological studies. Breast Cancer Res.

[B35] Persengiev SP, Green MR (2003). The role of ATF/CREB family members in cell growth, survival and apoptosis. Apoptosis.

[B36] Hayakawa J, Mittal S, Wang Y, Korkmaz KS, Adamson E, English C, Ohmichi M, McClelland M, Mercola D (2004). Identification of promoters bound by c-Jun/ATF2 during rapid large-scale gene activation following genotoxic stress. Mol Cell.

[B37] Cha-Molstad H, Keller DM, Yochum GS, Impey S, Goodman RH (2004). Cell-type-specific binding of the transcription factor CREB to the cAMP-response element. Proc Natl Acad Sci USA.

[B38] Liang G, Hai T (1997). Characterization of human activating transcription factor 4, a transcriptional activator that interacts with multiple domains of cAMP-responsive element-binding protein (CREB)-binding protein. J Biol Chem.

[B39] Fronsdal K, Engedal N, Slagsvold T, Saatcioglu F (1998). CREB binding protein is a coactivator for the androgen receptor and mediates cross-talk with AP-1. J Biol Chem.

[B40] Maekawa T, Shinagawa T, Sano Y, Sakuma T, Nomura S, Nagasaki K, Miki Y, Saito-Ohara F, Inazawa J, Kohno T (2007). Reduced levels of ATF-2 predispose mice to mammary tumors. Mol Cell Biol.

[B41] Maekawa T, Sano Y, Shinagawa T, Rahman Z, Sakuma T, Nomura S, Licht JD, Ishii S (2008). ATF-2 controls transcription of Maspin and GADD45 alpha genes independently from p53 to suppress mammary tumors. Oncogene.

[B42] Callens N, Baert JL, Monte D, Sunesen M, Van Lint C, de Launoit Y (2003). Transcriptional regulation of the murine brca2 gene by CREB/ATF transcription factors. Biochem Biophys Res Commun.

[B43] Shen Q, Uray IP, Li Y, Krisko TI, Strecker TE, Kim HT, Brown PH (2008). The AP-1 transcription factor regulates breast cancer cell growth via cyclins and E2F factors. Oncogene.

[B44] Gong H, Guo P, Zhai Y, Zhou J, Uppal H, Jarzynka MJ, Song WC, Cheng SY, Xie W (2007). Estrogen deprivation and inhibition of breast cancer growth in vivo through activation of the orphan nuclear receptor liver X receptor. Mol Endocrinol.

[B45] Nguyen DV, Rocke DM (2002). Partial least squares proportional hazard regression for application to DNA microarray survival data. Bioinformatics.

[B46] Li H, Gui J (2004). Partial Cox regression analysis for high-dimensional microarray gene expression data. Bioinformatics.

[B47] Vijver MJ van de, He YD, van't Veer LJ, Dai H, Hart AA, Voskuil DW, Schreiber GJ, Peterse JL, Roberts C, Marton MJ (2002). A gene-expression signature as a predictor of survival in breast cancer. N Engl J Med.

[B48] Wang Y, Klijn JG, Zhang Y, Sieuwerts AM, Look MP, Yang F, Talantov D, Timmermans M, Meijer-van Gelder ME, Yu J (2005). Gene-expression profiles to predict distant metastasis of lymph-node-negative primary breast cancer. Lancet.

[B49] Yeap BB, Krueger RG, Leedman PJ (1999). Differential posttranscriptional regulation of androgen receptor gene expression by androgen in prostate and breast cancer cells. Endocrinology.

[B50] Carroll JS, Meyer CA, Song J, Li W, Geistlinger TR, Eeckhoute J, Brodsky AS, Keeton EK, Fertuck KC, Hall GF (2006). Genome-wide analysis of estrogen receptor binding sites. Nat Genet.

[B51] Chin K, DeVries S, Fridlyand J, Spellman PT, Roydasgupta R, Kuo WL, Lapuk A, Neve RM, Qian Z, Ryder T (2006). Genomic and transcriptional aberrations linked to breast cancer pathophysiologies. Cancer Cell.

[B52] Sotiriou C, Wirapati P, Loi S, Harris A, Fox S, Smeds J, Nordgren H, Farmer P, Praz V, Haibe-Kains B (2006). Gene expression profiling in breast cancer: understanding the molecular basis of histologic grade to improve prognosis. J Natl Cancer Inst.

[B53] Miller LD, Smeds J, George J, Vega VB, Vergara L, Ploner A, Pawitan Y, Hall P, Klaar S, Liu ET (2005). An expression signature for p53 status in human breast cancer predicts mutation status, transcriptional effects, and patient survival. Proc Natl Acad Sci USA.

[B54] Minn AJ, Gupta GP, Siegel PM, Bos PD, Shu W, Giri DD, Viale A, Olshen AB, Gerald WL, Massague J (2005). Genes that mediate breast cancer metastasis to lung. Nature.

[B55] Ein-Dor L, Kela I, Getz G, Givol D, Domany E (2005). Outcome signature genes in breast cancer: is there a unique set?. Bioinformatics.

[B56] Teschendorff AE, Journee M, Absil PA, Sepulchre R, Caldas C (2007). Elucidating the altered transcriptional programs in breast cancer using independent component analysis. PLoS Comput Biol.

[B57] Niida A, Smith AD, Imoto S, Tsutsumi S, Aburatani H, Zhang MQ, Akiyama T (2008). Integrative bioinformatics analysis of transcriptional regulatory programs in breast cancer cells. BMC Bioinformatics.

[B58] Smith DD, Saetrom P, Snove O, Lundberg C, Rivas GE, Glackin C, Larson GP (2008). Meta-analysis of breast cancer microarray studies in conjunction with conserved cis-elements suggest patterns for coordinate regulation. BMC Bioinformatics.

[B59] Tongbai R, Idelman G, Nordgard SH, Cui W, Jacobs JL, Haggerty CM, Chanock SJ, Borresen-Dale AL, Livingston G, Shaunessy P (2008). Transcriptional networks inferred from molecular signatures of breast cancer. Am J Pathol.

[B60] Bussemaker HJ, Li H, Siggia ED (2001). Regulatory element detection using correlation with expression. Nat Genet.

[B61] Liao JC, Boscolo R, Yang YL, Tran LM, Sabatti C, Roychowdhury VP (2003). Network component analysis: reconstruction of regulatory signals in biological systems. Proc Natl Acad Sci USA.

[B62] Gao F, Foat BC, Bussemaker HJ (2004). Defining transcriptional networks through integrative modeling of mRNA expression and transcription factor binding data. BMC Bioinformatics.

[B63] Boulesteix AL, Strimmer K (2005). Predicting transcription factor activities from combined analysis of microarray and ChIP data: a partial least squares approach. Theor Biol Med Model.

[B64] Porcher C, Swat W, Rockwell K, Fujiwara Y, Alt FW, Orkin SH (1996). The T cell leukemia oncoprotein SCL/tal-1 is essential for development of all hematopoietic lineages. Cell.

[B65] Robb L, Elwood NJ, Elefanty AG, Kontgen F, Li R, Barnett LD, Begley CG (1996). The scl gene product is required for the generation of all hematopoietic lineages in the adult mouse. Embo J.

[B66] Bernard O, Lecointe N, Jonveaux P, Souyri M, Mauchauffe M, Berger R, Larsen CJ, Mathieu-Mahul D (1991). Two site-specific deletions and t(1;14) translocation restricted to human T-cell acute leukemias disrupt the 5' part of the tal-1 gene. Oncogene.

[B67] Rabbitts TH (1994). Chromosomal translocations in human cancer. Nature.

[B68] Ono Y, Fukuhara N, Yoshie O (1997). Transcriptional activity of TAL1 in T cell acute lymphoblastic leukemia (T-ALL) requires RBTN1 or -2 and induces TALLA1, a highly specific tumor marker of T-ALL. J Biol Chem.

[B69] O'Neil J, Shank J, Cusson N, Murre C, Kelliher M (2004). TAL1/SCL induces leukemia by inhibiting the transcriptional activity of E47/HEB. Cancer Cell.

[B70] Park ST, Sun XH (1998). The Tal1 oncoprotein inhibits E47-mediated transcription. Mechanism of inhibition. J Biol Chem.

[B71] The Rosetta Inpharmatics Vant Veer Breast Cancer Data. http://www.rii.com/publications/2002/vantveer.html.

[B72] UCSC Genome Browser. http://hgdownload.cse.ucsc.edu/goldenPath/hg18/bigZips/.

[B73] Kel AE, Gossling E, Reuter I, Cheremushkin E, Kel-Margoulis OV, Wingender E (2003). MATCH: A tool for searching transcription factor binding sites in DNA sequences. Nucleic Acids Res.

[B74] Tibshirani R (1997). The lasso method for variable selection in the Cox model. Stat Med.

