# Supplementary figures and images for: Systematic identification of transcription factors associated with patient survival in cancers
Source: BMC Genomics. 2009 May 15;10:225. doi: 10.1186/1471-2164-10-225 (PMC2686740; doi:10.1186/1471-2164-10-225)

**(A)**

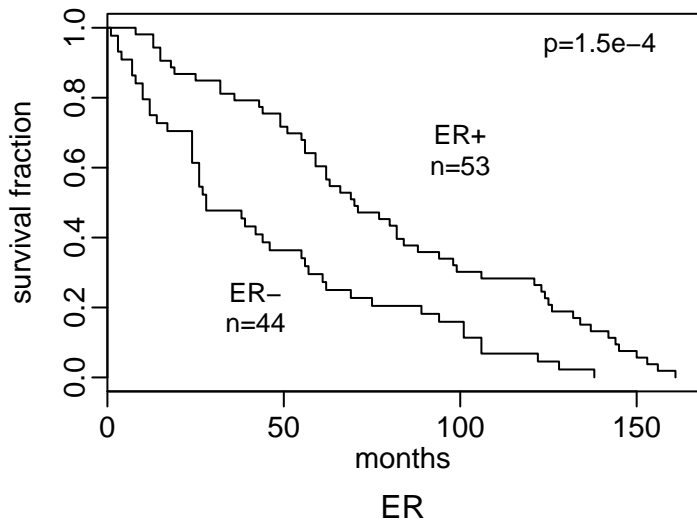

Supplement: Additional file 2 — Survival analysis of ER-positive and ER-negative breast cancer subgroups. The survival curves are estimated using the Kaplan-Meier method and the difference between subgroups is examined by the log-rank test. [file 1471-2164-10-225-S2.pdf]
